# 

**Published:** 1872-02

**Authors:** 


**PARTICULAR NOTICE.**

☞ *THE JOURNAL will not be sent, after the present number, to any who have not renewed their subscriptions.*

☞ **Remit $3 for the new volume, commencing January, 1872.**

